# Case report: Stent-first strategy as a potential approach in the management of malignant right-sided colonic obstruction with cardiovascular risks

**DOI:** 10.3389/fsurg.2022.1006020

**Published:** 2022-09-22

**Authors:** Tianyu Lin, Abdul Saad Bissessur, Pengfei Liao, Tunan Yu, Dingwei Chen

**Affiliations:** ^1^ Department of General Surgery, Sir Run Run Shaw Hospital, School of Medicine, Zhejiang University, Hangzhou, China; ^2^ Department of Surgical Oncology, Sir Run Run Shaw Hospital, School of Medicine, Zhejiang University, Hangzhou, China

**Keywords:** colonic obstruction, coronary stent, cardiovascular risk, self-expandable metallic stent, stent-first strategy, case report

## Abstract

In obstructing left-sided colonic or rectal cancer, endoscopic stent placement with the purpose of decompression and bridge to elective colon resection has been widely utilized and accepted. However, in malignant right-sided colonic obstruction, stent placement prior to colectomy is still highly controversial, due to lower clinical success and high anastomotic leak. We report a case of malignant right-sided colonic obstruction based on the radiological findings of irregular thickening of ascending colon wall and dilation of proximal large bowel on enhanced computed tomography scan. The 72-year-old woman presented with obvious abdominal distension. Due to concerning cardiovascular complications as intermittent chest pain and a long history of type 2 diabetes, a three-step therapeutic plan was instigated. Initially, a self-expandable metallic stent was placed palliatively to relieve the bowel obstruction. Consecutively, coronary angiography was performed, and two coronary stents were implanted to alleviate more than 80% stenosis of two main coronary arteries. One month later, laparoscopic radical resection of right colon and lymphadenectomy were successfully performed, with a blood loss less than 50 millimeters and a harvest of 29 lymph nodes, 1 being positive. The patient was discharged one week postoperatively with no complications, and received adjuvant chemotherapy one month later. During a follow-up of more than one year, the patient was in complete remission with no recurrence and cardiovascular events. In patients presenting with malignant right-sided colonic obstruction and peril of high cardiovascular risks, we propose colonic and coronary stent-first strategy to emergency surgery as a potential approach so as to ensure sufficient cardiovascular preparation improving perioperative safety. Moreover, the anatomical location of the tumor would be significantly achievable thus granting high-quality radical colon resection and lymphadenectomy.

## Core tip

Stenting has proven its effectiveness, predominantly in achieving a better quality of life, such as minimizing the use of colostomy, reducing medical costs and hospital stay. The role of stenting as a bridge to surgery for patients with left colonic obstruction should be debated from right-sided as stenting is strongly recommended as the alternative to emergency resection in left-sided malignant obstruction whereas its use in right-sided is still debatable and controversial. This case highlights the beneficial impact of stenting in right sided malignant colonic obstruction in a patient presenting with concurrent coronary artery stenosis.

## Introduction

Colorectal cancer is one of the most common cancers worldwide, ranking third in terms of incidence but as high as second with regard to mortality (1). Incidence rates tend to be higher in economically developed countries (2). Around one tenth of colon cancer initially present with bowel obstruction and present most commonly as abdominal pain, distention and obstipation (3). The risk of obstruction varies depending on the tumor location which is about 25% in the proximal colon compared to 75% in the left colon and colonic cancer causing obstruction tends to be at a more advanced stage (4).

Traditionally, emergency surgery, involving colectomy and colostomy has been the mainstay treatment for large bowel obstruction, a life-threatening condition (5). In the past two decades however, newer methodologies and treatment such as self-expandable metal stent (SEMS) or stoma construction (6) have proven to be more beneficial in patients with poor clinical condition and other contraindications to emergency surgery (7). However, detecting whether bowel obstruction requires definite emergency surgery is largely based on clinical signs and symptoms; high fever, tachycardia and peritonitis are suggestive of perforation or ischemia and demand urgent colectomy (3). As recommended by the European Society of Gastrointestinal Endoscopy (ESGE) Guideline 2020, colonic stenting is applicable for clinical symptoms and radiological signs of malignant large bowel obstruction, without any tip-off signs of perforation (8).

Despite SEMS followed by colectomy in patients with malignant colonic obstruction have had more favorable perioperative outcomes compared to urgent colectomy, the role of stent-first strategy in right colonic obstruction should be analyzed separately from that of left colonic obstruction, and remains unsettled and controversial (9). The use of stenting as a bridge to surgery has been extensively studied for malignant left sided obstruction, with several meta analyses and retrospective studies favoring the use of stent as an oncologically safe alternative over urgent colectomy (10–16). Interestingly, the latest update of ESGE Guideline 2020 strongly recommend the use of stenting as a bridge to surgery as an alternative to emergency resection in patients with curable left-obstructing colon cancer (8). In contrast, in patients with malignant right colonic obstruction, controversies arise and stent placement as a bridge to surgery is not yet the standard treatment approach and limited data are available on the safety and feasibility (17).

Herein, we present an elderly patient with malignant right-sided colonic obstruction and high cardiovascular risks in very poor clinical condition. The stent-first strategy brought sufficient time to manage the cardiovascular symptoms of the patient, thus improving perioperative safety. Moreover, high quality radical resection and extensive lymphadenectomy were achieved due to improvements in anatomy owing to stent placement.

## Case presentation

A 72-year-old female with obvious abdominal distention and pain for half a month was admitted at our hospital. Upon admission, she had intermittent chest pain in the inferior sternal region (pectoralgia) and tenderness in abdominal right lower quadrant. She had a long history of type 2 diabetes and underwent laparoscopic cholecystectomy 3 weeks prior to admission at a local hospital. Her abdominal symptoms did not alleviate and her medical condition deteriorated. She also complained about repeated chest distress. She had no significant personal and family history. Upon physical examination, the patient’s blood pressure was 143/76 mmHg, respiratory rate was 12 bpm, pulse rate was 90 bpm with a body temperature of 36.5° C. No cachexia or dehydration signs was observed. Her lung and heart auscultation were normal without apparent murmur or friction. Abdominal distention was obvious and tenderness in right lower quadrant upon palpation. The elevated laboratory findings are shown in Table 1, other laboratory evaluations were normal.

**Table 1:** Patient’s laboratory results of blood chemistry.

Parameter	Results	Reference value
White blood cell count (×10^9^/L)	13.3	3.5–9.5
Neutrophils (%)	84.0	40.0–75.0
c-reactive protein (CRP) (mg/L)	86.4	<6.0
Hemoglobin (g/L)	109	130–175
Total protein (g/L)	65.3	65.0–85.0
Albumin (g/L)	33.7	40.0–55.0
Glucose (mmol/L)	16.51	4.30–5.90
Sodium (mmol/L)	133	135–147
Ferritin (ng/ml)	358.40	12–150
Carcinoembryonic antigen (CEA)	1.82	0–5
CA125	46.91	<35.00

**Figure 1:**
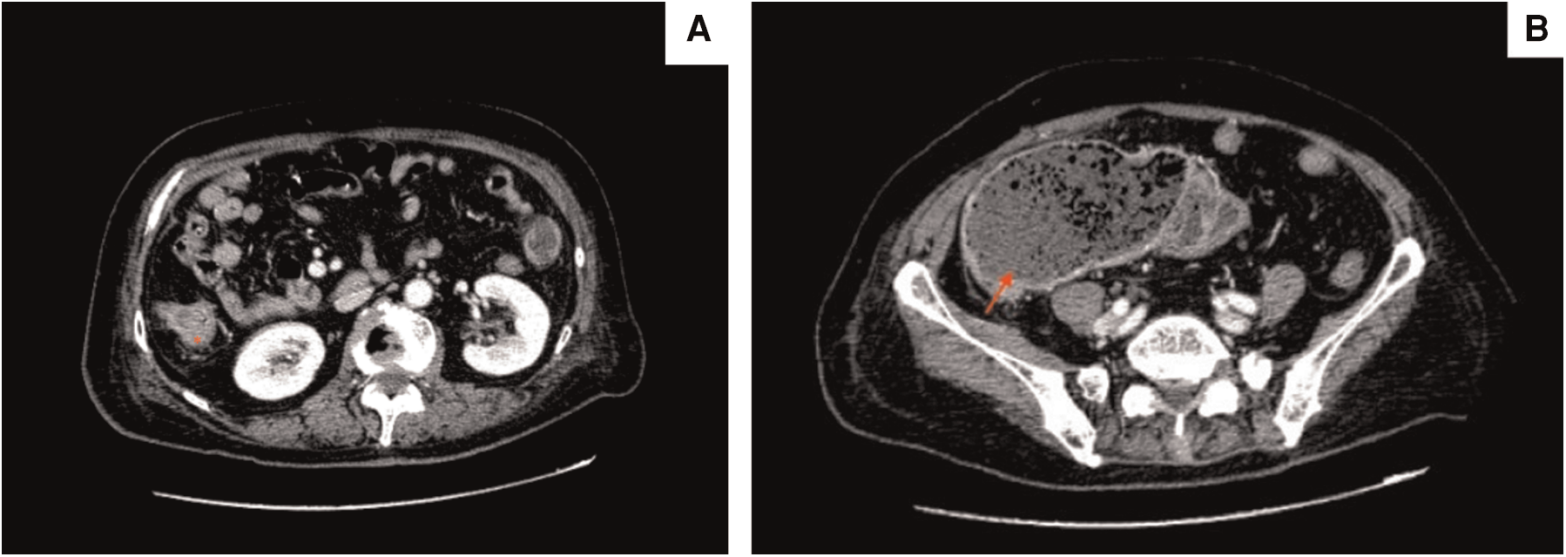
Ct scans showing (**A**) irregular thickening of ascending colon wall (marked by asterisk) and (**B**) proximal intestinal bowel distension (red arrow).

Enhanced abdominal computed tomography (CT) showed irregular thickening of ascending colon wall and proximal intestinal bowel dilation (Figure 1), suggesting malignant right-sided colonic obstruction. CT revealed no obvious ascites, liver metastasis or mesenteric lymph nodes enlargement. After carefully evaluating the patient’s symptoms given her advanced age, cardiovascular symptoms (pectoralgia and chest distress) and more than 10 years’ history of diabetes, a multidisciplinary team consultation consisting of gastrointestinal surgeons, cardiologists, an endoscopist and anesthesiologist devised a three-step plan: (1) SEMS to relieve bowel obstruction, (2) coronary angiography and placement of coronary stent, and (3) right hemicolectomy 1 month later.

**Figure 2:**
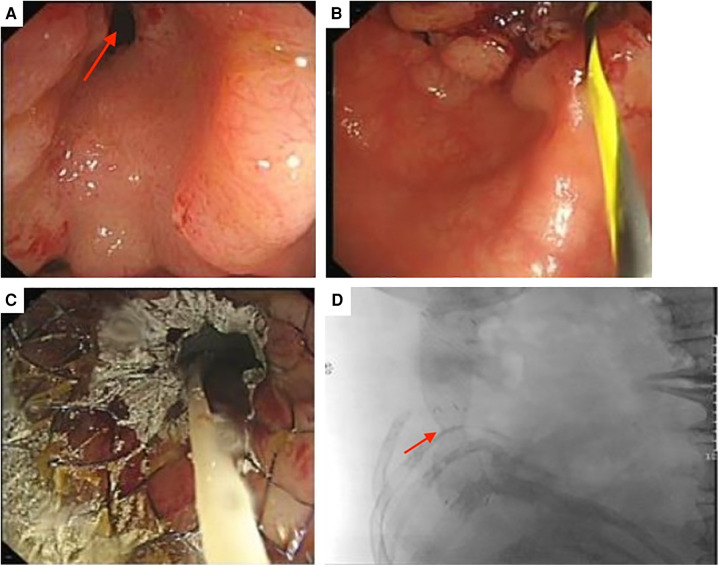
(**A**) Endoscopy revealing a narrowed intestinal lumen (red arrow). (**B**) Prior to placement of SEMS (**C**) successful stent placement relieving intestinal obstruction (**D**) x-ray showing location of stent and significant relief of intestinal obstruction.

SEMS was placed through endoscopy (Figure 2) and bowel obstruction was relieved. During this primary procedure, biopsy of the tumor sample was pathologically examined and indicated tubular villous adenoma, low-grade intraepithelial neoplasia and some high-grade intraepithelial neoplasia. Due to superficial sampling, no submucosal infiltration was observed, but adenocarcinoma could not be excluded.

**Figure 3:**
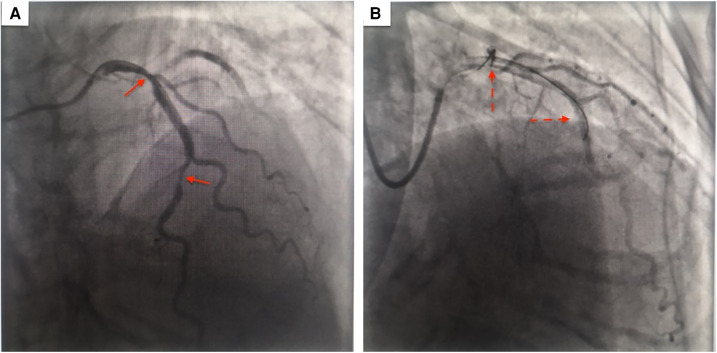
(**A**) Coronary angiography revealing 80% and 90% stenosis in the middle and distal left anterior descending branch of the heart (red arrow) (**B**) stenosis alleviated after placement of 2 stents (red dotted arrow).

**Figure 4:**
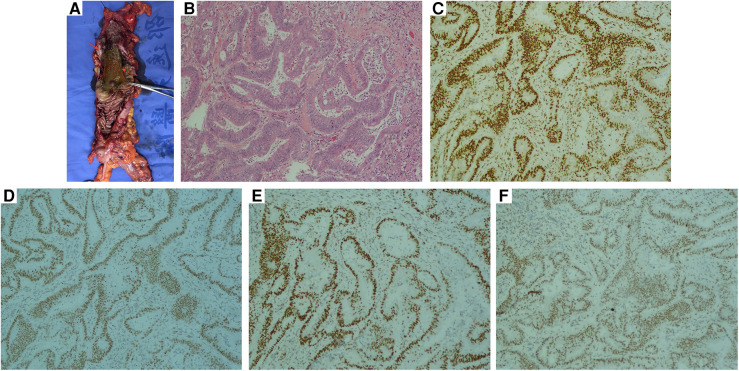
(**A**) A 6.5 cm ×  5 cm tumor infiltrating the subserosal layer (red arrow). The previously placed stent can be observed (red dotted arrow) (**B**) histologic findings of right colonic adenocarcinoma following hematoxylin and eosin stain. Immunohistochemical positivity of (**C**) MLH1(+) (**D**) MSH2 (**E**) MSH6 (**F**) PMS2 (**B–F**, magnification ×200).

Consecutively, coronary angiography revealed 80% and 90% stenosis in the middle and distal left anterior descending branch of the heart (Figure 3A) respectively and were subsequently alleviated by two coronary stents (Figure 3B). One month later, the patient underwent laparoscopic right hemicolectomy and wide mesenteric excision. During the surgery, multiple adhesions were observed in the abdominal cavity, and no obvious metastasis was noted. Blood loss was less than 50 milliliters. The tumor size was 6.5 × 5 cm (Figure 4A), infiltrating into the subserosal layer. Postoperative pathological examination revealed highly to moderately differentiated adenocarcinoma (Figure 4B), and 1 positive lymph node (1+/29). Immunohistochemistry findings revealed MLH1(+), MSH2(+), MSH6(+), PMS2(+) (Figures 4C–F). The patient was staged as T3N1aM0 (Stage IIIB, Eighth Edition AJCC).

The patient was discharged 1 week postoperatively with no complications. One month later, the patient received adjuvant chemotherapy for 6 cycles with each regimen cycle consisting of rituximab 600 mg IVGTT D1, oxaliplatin 130 mg IVGTT D1, epirubicin 90 mg IVGTT D1, vincristine 2 mg IVGTT D1 and dexamethasone 15 mg IV D1-3. One and a half year later, the patient showed no signs of recurrence on follow-up.

## Discussion

Colorectal cancer ranked third regarding incidence and second in terms of leading cause of cancer death worldwide as of 2020, accounting for 1.9 million new cases and an estimate of over 930,000 deaths (1). The burden of colorectal cancer has increased since 2012, where 1.35 million new cases were reported with an estimate of 700,000 deaths (18) and is expected to increase by 60% with more than 2.2 million new cases annually and 1.1 million deaths by 2030 (19). Occurring in about 15% of colon cancer patients, large bowel obstruction has been associated with increasing postoperative complications, mortality and a poor 5-year survival rate (20–22).

Traditionally, emergency surgery has been the mainstay treatment for malignant large bowel obstruction. However, anastomotic leak is a major postoperative burden of emergency surgery (12, 17). Reported in the early 90s by Dohmoto et al. (23) and Tejero et al. (24), stent placement as a bridge to surgery, to which we refer as stent-first strategy, has revolutionized the treatment of colon cancer, not only palliatively but also as a preoperative treatment (bridge to surgery) before final treatment approach (colectomy or colostomy) in suitable patients (25), as observed in our case. Over the years, stenting has proven its usefulness and effectiveness, predominantly in achieving a better quality of life, such as minimizing the use of temporary or permanent colostomy as well as reducing medical costs and hospital stay (12, 26–29). Moreover, in patients with inoperable tumors or presenting at advanced stages, stenting grants earlier initiation of neoadjuvant chemotherapy (30). As observed in our case, stenting also advocates minimally invasive surgery such as laparoscopic colectomy (31), thereby reducing postoperative complications and hospital stay.

However, the role of stent-first strategy or stenting as a bridge to surgery for patients with left colonic obstruction should be debated from right colonic malignant obstruction. The use of stents in left colonic cancer or rectal cancer has been extensively studied and reported favorably in several meta-analyses involving over 30 studies (11, 12) and retrospective case series (10). It is noteworthy to mention that stenting as a bridge to surgery is strongly recommended as the alternative to emergency resection in left sided malignant obstruction as per the 2020 latest guideline of ESGE (8), an upgrade compared to the 2014 Guideline by Van Hooft et al. (32) where stent placement as a bridge to surgery was still not recommended, thus proving its efficiency. Nonetheless, pertaining to malignant right-sided colonic obstruction, stent-first strategy is still controversial and currently not accepted as the standard treatment, prioritizing emergency resection and primary anastomosis (33). Limited data are accessible about the effectiveness and feasibility of stenting in right-sided colonic obstruction (17, 34).

Previous studies demonstrating the astounding safety of anastomosis in emergency resection without mechanical bowel preparation obstruction could advocate for the lack of studies of stenting in malignant right-sided colonic (33, 35). Moreover, stent placement for right colonic obstruction is technically more challenging and arduous than left sided occlusions (9), originating from the difficulty of passaging through obstructive lesions. However, recent studies (36–38) have reported a higher risk of anastomotic leak and mortality rate following emergency colectomy for malignant obstruction. These concerns elicited surgeons to find a safer approach such as stent-first strategy. A recently published meta-analysis concluded a significantly higher rate of successful primary anastomosis in stenting than the emergency surgery group (39). Stent placement also favors mechanical bowel preparation finally resulting in end-to-end anastomosis without use of colostomy and stoma (40), thereby significantly improving quality of life. In addition, since obstructing colon cancer specifically affect elderly patients presenting with other morbidities (3, 41, 42), stent-first strategy could be life-saving, as observed in our patient.

Recent studies supported the outstanding virtue of stent-first strategy in malignant right-sided colonic obstruction. In a nationwide database study of 1,500 patients with malignant right colon obstruction, stenting followed by colectomy compared to emergency colectomy provided more beneficial perioperative outcomes such as lower morbidity, reduced need of stoma, reduced postoperative hospital stay and lesser surgical site infection (9). In a retrospective study of 98 patients who underwent stent-first strategy was associated with better operative and oncological outcomes including likelihood of laparoscopic approach, less estimated blood loss, faster post-operative restoration of gastrointestinal function, lower post-operative and wound-related complication rate (17). In a small sized study by Ji et al. (31) assessing 14 patients who successfully underwent stent placement, the rate of laparoscopic approach was higher in the stent-first group compared to the emergency group. In addition, time to resume oral food intake was shorter in the stent group. Despite its controversial use in right-sided colonic obstruction, a recent meta-analysis confers that stenting as a bridge to surgery results in a reduction of postoperative complications and mortality for right-sided malignant large bowel obstruction than emergency resection (43).

Timing of surgical resection following stent placement varies. The ESGE recommends approximately 2 weeks until resection (8). The above-mentioned retrospective study of 98 patients revealed a median interval time of 18.5 days between stenting and surgery (17), whereas the mean time for the small sized study of 14 patients was 7 days (31). In our case, since coronary stents were placed in conjunct with colonic stent, an interval of 1 month proved convenient. Nevertheless, primary colectomy after successful stent placement should be not be overly delayed as the major complications of stenting are recurrent colonic obstruction, stent migration or perforation (44). Additionally, the possible risk factors such as stent-related perforation, higher recurrence rates, permanent stoma, technical and clinical failure rates should be discussed with the patient prior to stent placement (8).

The drawbacks of stent-first strategy should not be overlooked. Previous studies concluded a higher rate of perineural invasion (45), lymphatic invasion (46) and increase in tumor cells dissemination (47) following stent placement. Moreover, a study by Maruthachalam et al. (48) revealed an increase in circulating cytokeratin 20 mRNA levels following stent placement. Stent placement also carries the risk of perforations (49) and has been associated with a worse survival rate (50), although a recent trial found no significant difference in overall survival and disease free survival between stent as bridge to surgery and emergency surgery at a minimum follow up of 3 years (51). Thus, stent placement has been recommended only in high-risk patients with an ASA score of 3 or higher and in patients older than 70 (32). Noteworthy, given that different studies brought different results and conclusions, stenting should be performed by endoscopist with adequate expertise (39). This could be a possible hypothesis as to why different studies have different conclusions.

In a nutshell, stent-first strategy was beneficial to this patient for 2 main aspects: (1) After bowel decompression, enough time was allocated for sufficient cardiovascular optimization, especially improvement of coronary stenosis, increasing anesthetic safety and reducing perioperative cardiovascular events including ischemia, myocardial infarction and lethal arrhythmia. Secondly, a 1-month interval between stenting and hemi-colectomy, tissue edema was resolved and laparoscopic approach deemed successful, increasing not only short term outcomes but also favoring oncological outcomes.

## Conclusion

In patients with malignant right-sided colonic obstruction and cardiovascular risk, stent-first strategy had the upper hand of allocating enough time for cardiovascular preparation, and improving perioperative safety. Moreover, better anatomical conditions were achieved due to stent placement, thus favoring minimally invasive surgery, which decrease postoperative complications and facilitate early recovery.

## Data Availability

The original contributions presented in the study are included in the article/Supplementary Material, further inquiries can be directed to the corresponding author/s.
